# A Review of Hepatoprotective Plants Used in Saudi Traditional Medicine

**DOI:** 10.1155/2014/890842

**Published:** 2014-12-18

**Authors:** Abdulrahman K. Al-Asmari, Abdulrahman M. Al-Elaiwi, Md Tanwir Athar, Mohammad Tariq, Ahmed Al Eid, Saeed M. Al-Asmary

**Affiliations:** ^1^Department of Research, Prince Sultan Military Medical City, P.O. Box 7897 (775s), Riyadh, Saudi Arabia; ^2^Department of Urology, Prince Sultan Military Medical City, P.O. Box 7897 (775s), Riyadh, Saudi Arabia; ^3^Department of Clinical Pharmacy, Prince Sultan Military Medical City, P.O. Box 7897 (775s), Riyadh, Saudi Arabia; ^4^Department of FCM, Prince Sultan Military Medical City, P.O. Box 7897 (775s), Riyadh, Saudi Arabia

## Abstract

Liver disease is one of the major causes of morbidity and mortality across the world. According to WHO estimates, about 500 million people are living with chronic hepatitis infections resulting in the death of over one million people annually. Medicinal plants serve as a vital source of potentially useful new compounds for the development of effective therapy to combat liver problems. Moreover herbal products have the advantage of better affordability and acceptability, better compatibility with the human body, and minimal side effects and is easier to store. In this review attempt has been made to summarize the scientific data published on hepatoprotective plants used in Saudi Arabian traditional medicine. The information includes medicinal uses of the plants, distribution in Saudi Arabia, ethnopharmacological profile, possible mechanism of action, chemical constituents, and toxicity data. Comprehensive scientific studies on safety and efficacy of these plants can revitalise the treatment of liver diseases.

## 1. Introduction

Since the dawn of history plants have played an important role in the treatment of human ailments. By trial and error the ancient population was relieving their sufferings by using herbs in a very primitive way. The history of many drugs which are in practice today could be traced back to the Hellenic civilization; drugs like castor oil, opium, olive, anise, peppermint, saffron, henbane, acacia, and yeast are mentioned in Egyptian Ebers Papyrus (1500 B.C.). Babylonians and Assyrians have mentioned a large number of herbal medicines, for example, coriander, cinnamon, liquorice, and so forth. Thya, written by the Chinese physician Chou Kung, in about 1100 B.C., describes the use of a number of plant drugs. The books of Sustruta, written in India at the beginning of the Christian era, describe some seven hundred herbal medicines. According to the recent analysis, man is recycled plant and plants fulfill a variety of human needs, as they are the source of nourishment, health, and pleasure [1]. Even the lowest form of plant life can be vital, and penicillin is only one—no doubt—the most famous antibiotics.

### 1.1. Greeko-Arab System of Medicine

Greeko-Arab system of medicine is a well-documented system of traditional medicine which was originated by Greek physicians and philosophers and enriched by Arabs. It was the work of the Greek philosopher-physician Hippocrates (460–377 B.C.), who freed herbal supplements from the realm of superstition and magic and gave it the status of science. He considered illness to be natural rather than a supernatural phenomenon, and he strongly suggested that herbal supplements should be administered without ritual ceremonies or magic [1]. After Hippocrates, Galen (131–200 A.D.) stands out for his contribution to traditional medicine. Galen introduced and practiced herbal medicine in preIslamic Egypt, serving as royal court physician to the king of Egypt [2]. Under his patronage, hundreds of new herbal supplements were researched, experimented, and developed for the treatment of almost all types of diseases. Galen's contributions in herbal remedies is highly regarded, even today the term galenical is applied to simple vegetable extractives. Arabs like Rhazes (850–932 A.D.), Avicenna (980–1037 A.D.), Al-Bitar (1180–1248 A.D.), and Al-Antaki (1510–1587) constructed an imposing edifice of Arab traditional medicine. “Avicenna” (the western name for Abu Sina), an Arab philosopher and physicist who wrote “Kitab-al-shifa” (The Canon of Medicine), is highly noteworthy. According to Greeko-Arab system of medicine, disease is a natural process resulting from the imbalance of various humors in the body. The humoral theory presupposes the presence of four humors: dam (blood), balgham (phlegm), safra (yellow bile), and sauda (black bile) in the body. The temperaments of persons are expressed by the words sanguine, phlegmatic, choleric, and melancholic according to the preponderance of the following four humors in their body, namely, blood, phlegm, yellow bile, and black bile, respectively. The humors themselves are assigned temperaments. Blood is hot and moist, phlegm is cold and moist, and yellow bile is hot and dry. According to the Greeko-Arab system herbs may restore humor imbalance and cure the diseases [3].

Greeko-Arab physicians identified the liver as one of the three principal organs of the body, along with the heart and the brain. According to Galen the liver is the “master organ” of the human body, arguing that it emerges before all other organs in the fetus formation. In his book entitled “On the Usefulness of the Parts of the Body” Galen described the liver as warm and moist organ involved in blood formation and principle instrument of sanguification. According to Avicenna liver is “the seat of nutritive or vegetative faculties” and “the seat of manufacture of the dense part of the humors”. According to Arab physicians, malfunction of liver may lead to a variety of diseases which may be corrected by appropriate herbal intervention.

### 1.2. Liver Diseases and Their Global Burden

Liver is the largest and most vital organ of the human body. Besides its crucial role in the metabolism of nutrients, liver is responsible for biotransformation of drugs and chemicals thereby protecting body against toxic foreign materials. In this process the liver is exposed to high concentration of toxic chemicals and their metabolites which may cause liver injury. There are more than hundred well known liver diseases with diversified etiopathology. The most frequent causes of hepatic disease include infectious agents (especially hepatitis viral A, B, and C), obesity related fatty liver disease, xenobiotics (alcohol, drugs, and chemicals) induced liver injury, inherited and genetic defects related liver diseases, autoimmune hepatitis, liver cirrhosis, and primary or secondary liver cancer.

Liver diseases are one of the leading causes of morbidity and mortality across the world. Around 1.3 million deaths worldwide are due to chronic viral hepatitis. Many clinic-led researchers have found that liver related mortality is as high as fourth for some age group and eighth overall. According to WHO estimates, about 1.4 million cases of hepatitis A occur annually and 2 billion people worldwide are infected with the hepatitis B virus. About 350 million live with chronic infection and 600,000 persons die each year due to the acute or chronic consequences of hepatitis B. About 130–170 million people are chronically infected with hepatitis C virus, and more than 350,000 people die from hepatitis C-related liver diseases each year [4]. The recent statistics clearly show that global burden of liver disease has increased over time with a huge impact on overall world population.

### 1.3. A Tilt towards Herbal Drugs

The treatment options for common liver diseases are limited due to the lack of hepatoprotective drugs in allopathic medicine. Moreover therapies developed along the principle of western medicine are often limited in efficacy, carry the risk of adverse effects, and are often too costly, especially for the habitants of developing world. For example, the effectiveness of treatments such as those using corticosteroids and interferon is inconsistent, carried the risk of adverse events, and is often too costly [5]. On the other hand plant derived compounds are easily accessible and affordable. There is a deep belief that herbal remedies symbolize safety because they are “natural” and fit into the image of a gentle and, therefore, harmless alternative to synthetic drugs. No doubt that herbs are staging a comeback and herbal “renaissance” is happening all over the world. Several recent surveys from Europe and the United States have demonstrated a sharp rise in the popularity and use of botanical drugs within a few years, with up to 65% of liver patients taking herbal preparations. The fact is that reliable hepatoprotective drugs are explicitly inadequate, and the search for natural herbal drugs has intensified in the recent decades.

In this review we summarized the scientific data published on thirty-five hepatoprotective plants used in Saudi Arabian traditional medicine, description of the plants and their distribution in Saudi Arabia, medicinal uses, experimental pharmacological studies, possible mechanism of action, chemical constituents, and toxicity studies.

## 2. Methodology

A list of hepatoprotective plants used in Saudi Arabia was prepared based on a nationwide survey of herbal drug used in traditional medicine for liver ailment by

- interviewing the patients visiting primary care centres of military hospitals of different regions of Saudi Arabia,
- review of traditional medicinal books/publications and folklore information.

A thorough of survey of literature on the pharmacological profile of these plants was undertaken to collect the published data for the period between 1975 and 2014 AD by using “Pubmed” and “Google Scholar” search engines. Attempt was made to determine if these plants have been tested for hepatoprotective activity using well-established experimental models including carbon tetrachloride (CCl_4_), thioacetamide, paracetamol, ethanol, and morphine induced liver damage. The liver enzymes including aspartate transaminase (AST), alanine transaminase (ALT), alkaline phosphatase (APT), total protein (TP), and albumin (Alb) were used as a marker of liver injury. Literature search also included reversal of toxin induced histopathological changes by plant drugs.

An attempt has been made to illustrate possible mechanism of hepatoprotective herbs with special reference to their antioxidant (ability to normalize oxidative stress markers) and inflammatory mediators. Available data about the chemical constituent of the hepatoprotective plants and their toxicity has also been presented.

Briefly, this review summarises the information about 35 hepatoprotective herbal drugs used in Saudi traditional medicine (Table 1) for the treatment of liver diseases including their botanical name, family, and part of the plant used, distribution of plants in Saudi Arabia, and their use in traditional medicine. The results of hepatoprotective studies on each plant, possible mechanism of action, and their chemical composition and toxicity data have been presented.

## 3. Results

### 3.1. *Apium graveolens* Linn

*Apium graveolens* Linn. (family: Apiaceae) locally known as “Karfas” is a biennial or perennial glabrous herb with a heavy aromatic smell found in Najd region of Saudi Arabia [6]. Seeds of* A. graveolens* have been widely used in traditional medicine for the treatment of liver and spleen disorders, jaundice [7], rheumatism, gout, and other inflammatory diseases [8, 9].

The hepatoprotective activity of the methanolic extract of* A. graveolens* seed has been studied against CCl_4_ [10, 11] and paracetamol [11, 12] induced liver damage.* A. graveolens* extract dose dependently attenuated the toxin induced biochemical (serum AST, ALT, APT, TP, and albumin) and histopathological changes in liver tissues. The protective activity of* A. graveolens* was comparable with silymarin a well-established hepatoprotective herbal drug [10, 12]. Acute toxicity studies on* A. graveolens* extract in rats showed no adverse symptoms. Lethal dose in 50% of rats (LD_50_) was found to be of 7.5 g/kg body weight (b.w.) clearly suggesting its large margin of safety [12]. Chronic toxicity studies on the extract also revealed no delirious effect or mortality over a period of 14 days [13].

The hepatoprotective effect of* A. graveolens* Linn. may be attributed to its anti-inflammatory [14] and antioxidant activities [15]. Phytochemical screening showed the presence of flavonoids, tannins, volatile oils, alkaloids, sterols, and triterpenes. Detailed chemical studies also showed the presence of limonene, p-dimethyl styrene, N-pertyl benzene, caryophyllene, *α*-selinene, N-butyl phthalide, and sedanenolide [13].

### 3.2. *Artemisia scoparia* Waldst.et Kit

*Artemisia scoparia* Waldst.et Kit. (family: Compositae) locally known as “Baeiteran/A'weejan” is an annual herb mostly found in eastern and Najd region of Saudi Arabia [6]. The aerial part of* A. scoparia* has long been used in folk medicine for the treatment of jaundice and other liver disorders [16, 17].

The hepatoprotective activity of hydroalcoholic extract of aerial parts* A. scoparia* was investigated against CCl_4_ [18, 19] and paracetamol [20] induced liver damage. The extract dose dependently attenuated hepatotoxin induced biochemical parameters (rise in serum AST and ALT) and prolongation of phenobarbital induced sleeping time clearly indicating its hepatoprotective action. Hepatotoxins like CCl_4_ and paracetamol significantly reduced the activity of drug metabolizing enzymes in liver, leading to the slowing of drug metabolism resulting in increased level of drugs such as barbiturates which results in prolongation of their pharmacological activity (sleeping time). Reversal of barbiturate induced sleeping time suggests hepatoprotective effect of* A. scoparia* [18].* A. scoparia* also has a potent choleretic activity as evident from significant increase in bile volume, bile acid, and bile salt [21]. Recent pharmacological studies also showed anti-inflammatory [22] and antioxidant [23] activities of* A. scoparia* which may contribute to its hepatoprotective activity. Although the plant is recognised as antihelmintic, its mammalian toxicity is negligible [24]. Some cases of dermatitis and allergic reaction have been reported [25]. Phytochemical studies on aerial part of* A. scoparia* showed the presence of hyperin, eupafolin, pedalitin, 5,7,2′,4′-tetrahydroxy-6,5′-dimethoxyflavone, camphor, 1,8-, beta-caryophyllene, cirsilineol, cirsimaritin, arcapillin, and cirsiliol [26].

### 3.3. *Bacopa monnieri* Linn

*Bacopa monnieri* Linn. (family: Plantaginaceae) locally known as “Farfakh” is a small creeping glabrous perennial herb. In Saudi Arabia the plant grows in Tabuk, Al Jauf, Sakakah, northern Hejaz, and eastern region [6].* B. monnieri* is largely treasured as a revitalizing herb. In traditional medicine it has been used for more than 3000 years for the treatment of jaundice, liver diseases, spleen disorders, and digestive problems [27, 28].

Hepatoprotective activity of ethanolic extract of whole plant of* B. monnieri* has been studied against nitrobenzene [29] and morphine [30] induced liver toxicity. The extract significantly attenuated hepatotoxin induced changes in biochemical parameters (sera AST, ALT, and APT) and histopathological changes in liver tissues. Ethanolic extract of* B. monnieri* also showed significant antioxidant [30] and anti-inflammatory [31] activities, which may contribute to its hepatoprotective activity. Acute toxicity studies showed no deterious effect in pharmacological doses. The single dose LD_50_ was found to be 2400 mg/kg b.w. in rats. In a chronic toxicity study in rats,* B. monnieri* was found to be well tolerated up to the dose of 500 mg/kg b.w. for 3 months [32]. Phytochemical analysis on plant of* B. monnieri* showed the presence of alkaloid (brahmine), bacosides, nicotine, herpestine, D-mannitol, hersaponin, stigmosterol, beta-sitosterol, and bacosaponins [30, 33]. Bacoside, a major constituent of brahmi, has been shown to possess significant anticancer activity against liver tumors in rats [34].

### 3.4. *Balanites aegyptiaca* Linn

*Balanites aegyptiaca* Linn. (family: Zygophyllaceae) locally known as “Sidrul Kajjab” is a small shrub with thorn on stem. In Saudi Arabia, it is abundant in southern part of Hejaz ranging from Jeddah to Yemen border [6]. The bark, unripe fruits, and leaves of the* B. aegyptiaca* are used in folk medicine for the treatment of jaundice, liver disorders, and spleen problems [35].

The effect of ethanolic extracts of bark of* B. aegyptiaca* has been investigated against paracetamol [36] and CCl_4_ [37] induced hepatotoxicity in rats. The extract dose dependently attenuated the hepatotoxin induced biochemical (serum AST, ALT, ALP, and bilirubin) and histopathological changes in liver which was comparable with silymarin. The extract also reversed toxin induced prolongation of pentobarbital sleeping time in rats. The purified fractions of* B. aegyptiaca* possess significant antioxidant [38] and anti-inflammatory [39] activities which may contribute to its hepatoprotective action. Administration of 5% seed oil in diet produced mild toxicity in rats [40]. Phytochemical studies on* B. aegyptiaca* showed the presence of flavonoids, saponins, quercetin 3-glucoside, quercetin-3-rutinoside, 3-glucoside, 3-rutinoside, 3-7-diglucoside, and 3-rhamnoglucoside [41].

### 3.5. *Beta vulgaris* Linn

*Beta vulgaris* Linn. (family: Amaranthaceae) locally known as “shahya” is an annual or biennial herb found mostly in North Hejaz and Eastern Najd region of Saudi Arabia [6].* Beta vulgaris* is extensively cultivated as an article of food and the roots are used for the production of sugar. The plant root has been used in traditional medicine for a wide range of diseases including spleen and liver problems and inflammatory disorders [42–44].

Oral administration of the ethanolic extract of* Beta vulgaris* roots exhibited significant and dose dependent hepatoprotective activity against CCl_4_ induced liver damage in rats [45]. The hepatoprotective activity of* Beta vulgaris* may be attributed to its antioxidant [46] and anti-inflammatory [14] activities. The plant is safe to use even in large doses. Phytochemical studies on roots of* Beta vulgaris* Linn. have shown the presence of betaine, betacyanins, betaxanthins, oxalic acid, and ascorbic acid [47].

### 3.6. *Boerhavia diffusa* Linn

*Boerhavia diffusa* Linn. (family: Nyctaginaceae) locally known as “maddad” is a tall glabrous plant with a forked herbaceous stem widely distributed in Abha, Bisha, Najran, and Hejaz region of Saudi Arabia [6].* Boerhavia diffusa* has been widely used in traditional system of medicine for the treatment of jaundice and other liver diseases, internal inflammation, gall bladder problem, and spleen disorders [48].

Aqueous and ethanolic extracts of* B. diffusa* significantly attenuated acetaminophen [49] and ethanol [50] induced biochemical (rise of serum AST, ALT, APT, and bilirubin) and histopathological changes in liver suggesting its hepatoprotective action. The extract has been shown to possess significant antioxidant [49] and anti-inflammatory [51] activities which may contribute to its hepatoprotective activity. The oral LD_50_ for* B. diffusa* leaves in mice and rats was found to be 2000 mg/kg b.w. [52]. The aerial part of* B. diffusa* is a rich source of flavonoids, steroids, and alkaloids. Detailed phytochemical analysis showed the presence of campesterol, daucosterol, sitosterols, punarnavine, boeravinones A-F, borhavone, amino acids, lignans, and tetracosanoic, esacosanoic, stearic, and ursolic acids [48].

### 3.7. *Camellia sinensis* Linn

*Camellia sinensis* Linn. (family: Theaceae) locally known as “Shai.” The leaves and buds of this plant are used to produce the popular tea beverage. Our survey showed that* Camellia sinensis* is the second most commonly used herb by Saudi population for liver problems [53]. The decoction is used for obesity/weight loss, arthritis, and other inflammatory conditions and as anticancer [54].

The hepatoprotective activity of the aqueous extract of* C. sinensis* has been studied against experimentally induced liver damage in rats. The extract significantly attenuated CC1_4_ induced biochemical (serum ALT, AST, ALP, total protein, and albumin) and histopathological changes in liver [55]. Tea decoction has been shown to possess significant antioxidant, anti-inflammatory, and immunomodulatory activities [56, 57], which may contribute to its hepatoprotective activity. The antioxidant and anti-inflammatory activity of tea has been attributed to saponin contents of* C. sinensis* [58]. High doses of tea may cause convulsion/stimulation of central nervous system (CNS) due to its caffeine contents [59]. Some cases of green tea induced liver toxicity have been reported [60, 61]. Phytochemical studies on aerial part of* C. sinensis* have shown the presence of saponins, flavonoids, quercetine, quercitrin, rutin, catechin, caffeine, theophylline, and theobromine [62].

### 3.8. *Clitoria ternatea* Linn

*Clitoria ternatea* Linn. (family: Fabaceae) locally known as “Al-clitoria” is a perennial plant with big 5–7 elliptic to lanceolate leaflets abundant in southern Hejaz region of Saudi Arabia [6]. The leaves, seeds, and flowers are used in traditional medicine for liver diseases [63, 64].

Methanolic extract of* C. ternatea* (200 mg/kg) significantly attenuated CCl_4_ [65] and paracetamol [66] induced biochemical (serum ALT, AST, and bilirubin levels) and histopathological alterations in liver. “Ayush-Liv.04” a polyherbal formulation consisting of 20%* C. ternatea* leaves as one of its constituents also showed significant hepatoprotective activity against ethanol and CCl_4_ induced liver damage in rats [67].* C. ternatea* possess significant anti-inflammatory [68] and antioxidant [69, 70] activities which may contribute to its hepatoprotective effects. Roots of* C. ternatea* did not show any toxicological signs or deaths up to doses of 3000 mg/kg b.w. [71]. Phytochemical studies on leaves of* C. ternatea* showed the presence of flavonoids, saponins, tannins, glycosides, quercetin, steroids, taraxerol, taraxerone, ternatins, and taraxerol [66, 69].

### 3.9. *Commiphora opobalsamum* Linn

*Commiphora opobalsamum* Linn. (family: Burseraceae) locally known as Ood-e-Balsan, Behsan, or Balessan is medicinal plant with small, thorny tree which grows widely in Mecca region of Saudi Arabia. Local folk healer uses it for the treatment of stomach, jaundice and liver diseases, joint pain, and inflammatory disorders [72, 73].

The hepatoprotective activity of ethanolic extract of* C. opobalsamum* was studied using an experimental model of hepatotoxicity in rats [72]. The extract dose dependently protected liver against CCl_4_ induced biochemical (serum AST, ALT, and APT) and prolongation of the barbiturate sleeping time. The extract also showed significant antioxidant [72] and anti-inflammatory [74] activities which may contribute to its hepatoprotective effects. Even the large doses of ethanolic extract of* C. opobalsamum* did not show adverse effects in rats [75]. Phytochemical studies on aerial part of* C. opobalsamum* showed the presence of saponins, volatile oil, sterol and/or triterpenes, friedelin, flavonoids, mearnsetin, and quercetin [72].

### 3.10. *Curcuma longa* Linn

*Curcuma longa *Linn. (family: Zingiberaceae) locally known as “curcum” is a small rhizomatous perennial herb [6]. The genus named* Curcuma* is the latinized form of the Arabic Al-Kurkum. For over 4000 years, it has been widely used in Asian traditional medicine for loss of appetite, jaundice, liver problems, gall bladder disorders, and arthritis [76, 77]. Experimental studies have substantiated its use as hepatoprotective and hypolipidemic [78].

Hepatoprotective effect of turmeric has been attributed to its antioxidant [79] and anti-inflammatory [80] properties. Sodium curcuminate, a salt of curcumin, also exerts choleretic effects by increasing biliary excretion of bile salts, cholesterol, and bilirubin, supporting its use for the treatment of cholelithiasis. Toxicity studies on* C. longa *in animals showed no adverse effect up to 2.5 g/kg b.w. [81]. In humans, large doses may cause gastric irritation. The healing effect of* C. longa* is attributed to polyphenolic curcuminoids including curcumin I, curcumin II, and curcumin III [78].

### 3.11. *Eruca sativa* Mill

*Eruca sativa *Mill. (family: Cruciferae) locally known as “Jarjeer” is a hairy plant having oblong leaves grows in northern Hejaz, Najd, and eastern region of Saudi Arabia [6]. In Greeko-Arab medicine,* E. sativa* is considered as general tonic [82]. It has been used for treatment of liver and intestinal disorders [83].* E*.* sativa* has gained greater importance as a salad vegetable and spice, especially among Middle Eastern populations and Europeans. The leaves and seeds have been investigated for their hepatoprotective, anti-inflammatory, and antioxidant activities [84].

The ethanolic extract of* E. sativa *leaves and seeds showed significant hepatoprotective activity against CCl_4_ [84] and ethanol [85] induced liver injury. The* E. Sativa* extract also showed significant cytoprotective effect against liver cancer cells [86]. The hepatoprotective activity of* E. Sativa* may be attributed to its antioxidant [87] and anti-inflammatory [88] activities. It is an edible plant with no reported toxicity. Phytochemical studies on leaves of* E. sativa* have shown the presence of large amount of polyphenols, flavonoids, erucin, erysolin, glucosinolates, quercetins, erucic acid, and phenylethyl isothiocyanate [84, 86].

### 3.12. *Ficus carica* Linn

*Ficus carica *Linn. (family: Moraceae) locally known as “Hammat teen” is a shrub with milky big palmately lobed leaves found mostly in southern Hejaz and Najd region of Saudi Arabia [6]. The fig is cultivated as an edible fruit. The plant has been widely used in Greeko-Arab traditional medicine for the treatment of liver diseases, stomach ailments, digestive problems, obesity, and inflammatory disorders [89–92].

The hepatoprotective activity of various extracts of* F. carica *leaves and fruits have been experimentally confirmed against CCl_4_ [93, 94] and rifampicin [95] induced hepatotoxicity. The hepatoprotective activity of* F. carica* may be attributed to its marked anti-inflammatory [96] and antioxidant [97] activities.* F. carica* being an edible fruit is generally considered safe; however the unripe fruit may cause toxic effect and its sap may cause contact dermatitis [98]. Phytochemical studies on leaves and fruits of* F. carica *have shown the presence of flavonoids, vitamins, nicotinic acid, tyrosine, ficusin, bergaptene, stigmasterol, furocoumarin, psoralen, taraxasterol, beta-sitosterol, rutin, and sapogenin [93, 97].

### 3.13. *Grewia mollis* Juss

*Grewia mollis *Juss. (family: Malvaceae) locally known as “Nab'aaa” is a shrub/tree found mostly in Hejaz region of Saudi Arabia [6]. The leaves and bark of* G. mollis *have been used in traditional medicine for the treatment of liver diseases, abdominal problems, arthritis, and inflammatory conditions [99–101].

Methanolic extract of* G. mollis *leaves showed significant hepatoprotective activity against CCl_4_ induced liver injury [102].* G. Mollis* extract possesses significant antioxidant [102] and anti-inflammatory [99] activities which may contribute to its hepatoprotective effects. The pharmacological effect of* G. mollis *may be attributed to its steroidal and/or triterpenoidal constituent which have proven to be anti-inflammatory activity [99]. High doses of* G. mollis *stem bark may cause mild adverse effects including impairment of liver function [103]. Phytochemical studies on leaves of* G. mollis *has shown the presence of luteolin, tetrahydroxyflavone, 7*β*-hydroxy-23-enedeoxojessic acid, 7*β*-hydroxy-23-deoxojessic acid, *β*-sitosterol, and *β*-sitosterol-3-O-glucoside [99, 102].

### 3.14. *Grewia tenax* Forsk

*Grewia tenax* Forsk. (family: Malvaceae) locally known as “khaddar/shohat” is a glabrous shrub found in southern Hejaz region of Saudi Arabia [6]. In traditional medicine leaves, root, and fruits of* G. tenax *are used for the treatment of digestive diseases, liver disorders, jaundice, and inflammatory conditions [4, 104].

The administration of ethanol extract of* G. tenax *significantly restored CCl_4_ induced biochemical (serum AST, ALT, APT, TB, and gamma-glutamyl transferase) and histopathological changes in rats. Reversal of pentobarbital-induced prolongation of narcolepsy by the extract also suggested its hepatoprotective effect. The chronic administration of extract significantly reduced cholesterol, low-density lipoproteins, and triglycerides level [105]. The hepatoprotective effect of* G. tenax *is attributed to antioxidant [105] and anti-inflammatory [105, 186] properties. Experimental studies in mice showed no adverse effect except mild diarrhea in the high dose of 2 g/kg b.w. of ethanolic extract [105]. Phytochemical studies on plant of* G. tenax* have shown the presence of triacontan-1-ol, *α*-amyrin, *β*-amyrin, *β*-Sitosterol, lupenne, erythrodiol, betulin, and tetratriacont-21-ol-12-one [4, 105].

### 3.15. *Haloxylon salicornicum* Moq

*Haloxylon salicornicum *Moq. (family: Chenopodiaceae) locally known as “Armas” is a stout herb with green succulent branches distributed in all the regions of Saudi Arabia [6]. In Arabian Peninsula and other Asian countries* H. salicornicum *has been used for the treatment of jaundice, gall bladder stones, liver diseases, digestive problems, inflammatory disorder, and joint diseases [106–108]. Experimental studies confirmed hepatoprotective [109], anti-inflammatory [110, 187], and antiulcer [107] activity of* H. salicornicum*.

The ethanolic extract of* H. salicornicum* dose dependently attenuated CCl_4_ induced increase in liver enzymes and histological changes [109]. Recently Alqasoumi et al. [110] reported antioxidant and anti-inflammatory activities of* H. salicornicum* which may contribute to its hepatoprotective activity. The toxicity studies on* H. salicornicum *extract showed that, even in the high dose of 4 g/kg b.w., the extract did not produce any symptoms of toxicity or death in rats [110, 188]. Phytochemical studies on aerial part of* H. salicornicum *has shown the presence of alkaloids, tannins, saponin glycosides, 7-hydroxy-4-triacontanone, 24-hydroxy-4-octacosanone, 1-triacontanol, *β*-amyrin, 24-ethylcholesta-3,5-diene, 24-nor-12-ursene, *β*-sitosterol, ursolic acid, and *β*-sitosterol [109–111].

### 3.16. *Hypericum perforatum* Linn

*Hypericum perforatum *Linn. (family: Hypericaceae) locally known as “Ashba berfortum” is perennial herbs/shrubs with yellow flower. It is popularly known as St John's wart. It is found in southern region of Saudi Arabia [6]. The medicinal use of herbs is mentioned in the writing of famous Greeco-Arab physicians Istikoglou et al. [189]. Avicina, a famous Arab physician in his book “Canon of medicine” (“Al-Quanoon fil Tib” in Arabic), also described medicinal properties of this herb [190]. The traditional medicinal uses of* H. perforatum *include treatment of jaundice, liver diseases, gall bladder stones, rheumatoid arthritis, and other inflammatory conditions [112–114].

Ozturk et al. [113] reported the hepatoprotective effect of alcoholic extract of aerial part of* H. perforatum *extract. The extract significantly attenuated CCI_4_ and ethanol [112, 113] induced hepatic toxicity. Experimental studies also showed significant choleretic activity of* H. Perforatum* [112]. The protective action of* H. perforatum* has been attributed to its anti-inflammatory [191], antioxidant, and immunomodulating activities [192]. Acute toxicity studies in rodent showed no toxicity; however chronic administration for 2 weeks showed significant signs of erythema, dermal edema, alopecia, and changes in blood chemistry. The animals gained less weight as compared to control in chronically treated groups [193]. Phytochemical studies on plant of* H. perforatum *showed the presence of hypericin, pseudohypericin, hyperforin, adhyperforin, quercetin, hyperoside, rutin, campferol, myricetin, amentoflavone, kielcorin, and norathyriol [115].

### 3.17. *Juniperus procera* Hochst. ex Endl

*Juniperus procera *Hochst. ex Endl. (family: Cupressaceae) locally known as “Arar” is a long tree with needle like leaves found in Hejaz and southern region of Saudi Arabia [6]. The plant has long been used in Saudi traditional medicine for liver disease, jaundice, digestive problems, and inflammatory diseases [116]. The resin of* J. procera *in combination with honey is also used as cure for liver diseases and ulcers [115].

The ethanolic extracts of aerial part of* J. procera *showed significant hepatoprotective activity against CCl_4_ induced liver injury [117]. The hepatoprotective activity has been attributed to terpene contents of* J. procera *[116].* J. procera *possess significant antioxidant/free radical scavenging [194] and anti-inflammatory activities [195] which may contribute to its hepatoprotective activity. Acute and chronic toxicity studies revealed that the extract of* J. procera *is free from toxicity even in high dose [116]. Phytochemical studies on aerial part of* J. procera *showed the presence of terpenes, *β*-peltatin A, deoxypodophyllotoxin, and totarol [117].

### 3.18. *Lepidium sativum* Linn

*Lepidium sativum *Linn. (family: Cruciferae) locally known as “El-Rshad” is a fast-growing, edible herb with tangy flavour and aroma [6]. In traditional system of medicine various parts of plant have been used for the treatment of jaundice, liver problems, spleen diseases, gastrointestinal disorders, arthritis, and other inflammatory conditions [53, 118].

Hepatoprotective effect of methanolic extracts of* L. sativum *seeds was evaluated against CCl_4_ induced liver damage in rats. The extract dose dependently attenuated CCl_4_ induced rise in serum levels of AST, ALT, APT, and bilirubin suggesting its hepatoprotective activity [196]. Recently* L. sativum* has been shown to possess significant antioxidant [197–199] and anti-inflammatory [200] activities which may contribute to its hepatoprotective effect. In rats, up to 2% w/w of* L. sativum* in diet did not produce any toxicity, whereas 10% w/w showed mild toxicity [201]. Phytochemical studies on seed of* L. sativum *showed the presence of alkaloids, saponins, anthracene glycosides, carbohydrates, proteins, amino acids, flavonoids, and sterols [118].

### 3.19. *Moringa oleifera* Lam

*Moringa oleifera *Lam. (family: Moringaceae) locally known as “Ruwag” is a small, graceful, deciduous tree with sparse foliage [6]. The plant grows abundantly in many tropical and subtropical countries. Moringa is an ancient magic plant with a plethora of medicinal and nutritional value. The leaves, flowers, root, gums, fruit, and seed of* M. oleifera* have been extensively used in traditional medicine for the treatment of liver disease, lipid disorders, arthritis, and other inflammatory disorders [119–122].

Hepatoprotective effect of the ethanolic extract of* M. oleifera* leaves was studied against antitubercular drugs (isoniazid, rifampicin, and pyrazinamide) [202] induced liver damage as well as against cadmium induced hepatotoxicity in rats. Moringa extract significantly attenuated hepatotoxin induced biochemical (serum AST, ALT, APT, and bilirubin) and histopathological changes in liver. The hepatoprotective activity of* M. oleifera *was comparable with silymarin [203]. The extracts of* M. oleifera *leaves also showed significant antioxidant [204] and anti-inflammatory [120, 205] activities which may contribute to its hepatoprotective effect. The aqueous extract of* M. oleifera *is relatively safe with an LD_50_ value of 5 g/kg b.w. in mice [206]. Phytochemical studies of* M. oleifera *showed the presence of alkaloids, anthocyanins, *β*-carotene, protein, vitamin C, phenolics, calcium, iron, and potassium [122].

### 3.20. *Nigella sativa* Linn

*Nigella sativa *Linn. (family: Ranunculaceae) locally known as “Habbul-Barka” is a widely used medicinal plant throughout the world. According to Islamic and Arab literature, black seed of* N. sativa* is one of the most powerful herbal drugs used as liver tonics, digestive, anti-inflammatory, immunostimulant, and remedy for jaundice [123, 124].

Aqueous suspension of seeds powder of* N. sativa* showed significant hepatoprotective activity against CCl_4_ and ischemic-reperfusion induced liver injury [124, 207–211]. The anti-inflammatory [212–214] immunomodulating [215] antioxidant [216] activities of* N. sativa* may contribute to its hepatoprotective activity. The extracts and oil are relatively safe. The oral LD_50_ value of* N. sativa* fixed oil was found to be 28.8 mL/kg b.w. in mice [217]. Phytochemical studies on plant of* N. sativa *have shown the presence of thymoquinone, thymohydroquinone, dithymoquinone, p-cymene, carvacrol, and 4-terpineol [125].

### 3.21. *Peganum harmala* Linn

*Peganum harmala *Linn. (family: Nitrariaceae) locally known as “Harmal/Naqt” is a glabrous shrub found mostly in northern Hejaz and eastern Najd region of Saudi Arabia [6]. In traditional medicine* P. harmala* has been used for the treatment of jaundice, digestive disorders, liver diseases, and arthritis [126–129].

The hepatoprotective effect of ethanol and chloroform extracts of* P. harmala *seeds has been studied against thiourea [131] and CCl_4_ induced hepatotoxicity [218, 219] in rats. Both extracts dose dependently attenuated hepatotoxin induced biochemical (serum AST, ALT, and bilirubin) and histopathological changes suggesting its hepatoprotective activity. The extract also showed antioxidant [219] and anti-inflammatory [220] activities which may contribute to its hepatoprotective activity. Acute toxicity studies on the aqueous extract of* P. harmala* revealed that large doses may cause reversible tremors and convulsions in rats [221]. Oral LD_50_ in Wistar rats was found to be 2.70 g/kg b.w. In chronic studies aqueous extract of* P. harmala* administered orally for six weeks at doses of 1, 1.35, and 2 g/kg b.w. daily for 3-month period increased liver enzyme suggesting its hepatotoxicity. Histologic study also showed liver degeneration and spongiform changes in the central nervous system (CNS) in chronically treated rats [222]. Phytochemical studies on plant of* P. harmala* showed the presence of harmaline, harmine, harmalol, and tetrahydroharmine [130, 131, 223].

### 3.22. *Pergularia daemia* Forsk

*Pergularia daemia *Forsk. (family: Apocynaceae) locally known as “Ghalqa” is a climbing plant with thin glabrous leaves found in Najd region of Saudi Arabia [6]. In traditional system of medicine the whole aerial part of the plant is extensively used for the treatment of jaundice, liver diseases, and inflammatory disorders [132–134].

The ethanolic extracts of aerial parts of* P. daemia *dose dependently prevented the paracetamol [133] and CCl_4_ [134, 135] induced biochemical (serum AST, ALT, APT, and TB) and histopathological changes in the liver. Recent studies on* P. daemia *showed significant anti-inflammatory, antioxidant, and free radical scavenging activities [132–134] which may also contribute to its hepatoprotective activity. The ethanolic extract of* P. daemia *is relatively safe as it did not produce any toxicity up to a dose of 1.5 g/kg b.w. in mice [224]. Phytochemical studies on* P. daemia *have shown the presence of cardenolides, alkaloids, flavonoids, saponins, triterpenes, and steroidal compounds [134, 135].

### 3.23. *Petroselinum crispum* Mill

*Petroselinum crispum *Mill. (family: Umbelliferae) locally known as “Baqdunis” is a biennial herb widely grown in all the regions of Saudi Arabia [6].* P. crispum *has been used in Arab traditional medicine, for the treatment of inflammatory condition, liver diseases, constipation, flatulence, jaundice, colic pain, and rheumatism [136, 137].

Ethanolic extract of* P. crispum *leaves has been pharmacologically investigated for its hepatoprotective activity [138]. The extract dose dependently attenuated CC1_4_ induced increase in serum AST, ALT, ALP, and total bilirubin. The ethanolic extract of* P. crispum *leaves also showed significant anti-inflammatory [138] and antioxidant [225, 226] activities which may contribute to its hepatoprotective action. Although perfectly safe in pharmacological doses,* P. crispum* may be toxic in excess, especially when used as essential oil [227]. Phytochemical studies on* P. crispum *have showed the presence of flavones glycosides, apigenin-7-O-glucoside or cosmosiin, apigenin-7-O-apiosyl-O-glucoside/apiin, and the coumarin 2′′,3′′-dihydroxy furanocoumarin/oxypeucedanin hydrate [138–140].

### 3.24. *Phyllanthus maderaspatensis* Linn

*Phyllanthus maderaspatensis *Linn. (family: Euphorbiaceae) locally known as “Damabas” is a small branched shrub with scattered leaves and grows abundantly in eastern Najd and southern Hejaz region of Saudi Arabia [6]. In traditional medicine, sap and leaf decoction have been used as emetic and purgative; decoction of root is used for constipation, digestion, and abdominal pain. The aerial parts of plant have been used for treating liver disorders, rheumatism, and inflammatory diseases [141–143].

The hepatoprotective activity of whole plant extract of* P. maderaspatensis* has been investigated using several experimental models of hepatotoxicity [142, 228]. The extract significantly attenuated CCl_4_ induced biochemical (serum AST and ALT) and histopathological changes in liver. The hepatoprotective effect of* Phyllanthus* was comparable with silymarin [142].* P. maderaspatensis* showed strong antioxidant [229] and anti-inflammatory [230] activities which may contribute to its hepatoprotective activity.* P. maderaspatensis* is considered as safe in pharmacological doses [231]. Phytochemical studies on* P. maderaspatensis *showed the presence of carbohydrates, proteins, flavonoids, essential oil, and tannins. Seeds of* P. maderaspatensis* contain long chain fatty acids and *β*-sitosterol [142]. Defatted seed cake contains mucilage, which yields galactose, arabinose, rhamnose and aldobionic acid, niruriside, phyllanthin, hypophyllanthin, and cinnamoyl sucrose acetate [144].

### 3.25. *Pimpinella anisum* Linn

*Pimpinella anisum *Linn. (family: Umbelliferae) locally known as “Alyansoon” is one of the oldest known annual medicinal herbs with white flowers and small seeds. In Arab traditional medicine the plant is used as digestive, carminative, antispasmodic, and liver disorders [145, 146].

Diethyl ether extract of* P. anisum *seed has been investigated for its hepatoprotective activity in rats. The extract dose dependently attenuated CCl_4_ induced rise liver enzymes including AST and ALT [232].* P. anisum* possess significant antioxidant [233, 234] and anti-inflammatory [235] activities which may contribute to its hepatoprotective efficacy. Oral lethal dose of anise oil in human being ranges between 50 and 5000 mg/kg [236]. Essential oil of* P. anisum *has an LD_50_ value of 0.84 mL/kg b.w of mice whereas the fixed oil has an LD_50_ value of 3.15 mL/kg in mice [237]. Phytochemical studies on plant of* P. anisum *have shown the presence of volatile oils (anethole, eugenol, methyl chavicol, and estragole), fatty acids (palmitic, petroselinic, vaccenic, and oleic acids), and coumarins [147].

### 3.26. *Portulaca oleracea* Linn

*Portulaca oleracea *Linn. (family: Portulacaceae) locally known as “Rizlah” and “Farfahena” is an annual herb with branched stems found in Hejaz region and eastern part of Saudi Arabia [6]. The medicinal use of* P*.* oleracea* was known by Arabs from the time of Pharaohs [238]. It is used for the treatment of liver disorders, gastrointestinal problems, and inflammatory condition [82, 148].

The hepatoprotective activity of the aqueous and ethanolic extract of* P*.* oleracea* whole plant has been investigated by several investigators [148, 149, 239]. The extract significantly attenuated CCl_4_ induced rise in biochemical (serum AST, APT, TB, and total protein) and histopathological changes in liver. It also antagonised CCl_4_ and prolonged pentobarbitone induced sleeping time clearly suggesting significant hepatoprotective activity. The extracts of* P. oleracea *also showed significant antioxidant [240] and anti-inflammatory [241] activities which may contribute to its hepatoprotective activity. Methanolic extract of* P. oleracea *has an LD_50_ value of 1.8 g/kg b.w. in mice. In high doses the extract may cause kidney, lung, and liver toxicity in a dose dependent manner [242].* P. oleracea* contains several biologically active compounds that include, alkaloids, coumarins, flavonoids, cardiac glycosides, anthraquinone glycosides, alanine, saponins, tannins, and organic acids (free oxalic acids, cinnamic acids, caffeic acid, malic acids, and citric acids). Omega-3-acids, alpha-linolenic acid, vitamins, glutathione, glutamic acid, and aspartic acid containing *β*-sitosterol have also been found in various parts of plants [149–151].

### 3.27. *Rhazya stricta* Decne

*Rhazya stricta *Decne. (family: Apocynaceae) locally known as “harmal” is a perennial sand binding under shrub found in all regions of Saudi Arabia [6]. In the honor of Al-Rhazes, a leading scholar and physician of Arab and Islamic world, the plant was named as* Rhazya stricta*. In traditional medicine the plant is used for the treatment of inflammatory condition, stomach problems, and liver diseases [152–154].

Pretreatment with* R. stricta* significantly protected mice against paracetamol induced biochemical changes and prolongation of pentobarbitone induced sleeping time. The hepatoprotective effect of* R. stricta *was comparable with silymarin [36]. The extract of* R. stricta *leaves also showed significant antioxidant [154] and anti-inflammatory [243] activities which may contribute to its hepatoprotective activity. Ingestion in therapeutic doses is perfectly safe in human; however chronic administration of high doses in rats has shown variety of toxic effects including decrease in growth rate, dullness, and hepatonephrotoxicity [155, 244]. Phytochemical studies on* R. stricta *showed the presence of alkaloids (rhazimine, stemmadenine, vincadine, and rhazimanine), carboline, and flavonoidal glycoside [36, 154, 155].

### 3.28. *Smilax regelii *Killip and CV Morton

*Smilax regelii* Killip and CV Morton (family: Liliaceae) locally known as “Nabatul Fusaq” is a perennial, trailing vine with prickly stems [6]. The plant commonly known as sarsaparilla has been widely used for the treatment of liver diseases, arthritis, and other inflammatory conditions and as an immunomodulator in Greeko-Arab system of medicine [156–158]. Besides its medicinal use, sarsaparilla is often used as a flavouring agent in nonalcoholic drinks [245]. A decoction made from the roots is used as a vehicle in the preparation of syrups which have been reported to have cooling properties [246].

The hepatoprotective effect of the ethanol extract of roots of* S. regelii *has been studied in rats. Ethanolic extract of sarsaparilla significantly inhibited CCI_4_ induced rise in AST, ALT, and bilirubin, in serum in rats [247]. The extract showed strong antioxidant [247], anti-inflammatory [159], and immunomodulating [248] activities which may contribute to its hepatoprotective property. No known toxicity or side effects have been documented for sarsaparilla; however ingestion of large doses may cause gastric irritation [160]. Phytochemical studies on plant of* S. regelii *showed the presence of cetyl-parigenin, astilbin, beta-sitosterol, caffeoyl-shikimic acids, dihydroquercetin, diosgenin, engeletin, essential oils, epsilon-sitosterol, eucryphin, eurryphin, ferulic acid, glucopyranosides, isoastilbin, isoengetitin, kaempferol, parigenin, parillin, pollinastanol, resveratrol, rhamnose, saponin, sarasaponin, sarsaparilloside, sarsaponin, sarsasapogenin, shikimic acid, sitosterol-d-glucoside, smilagenin, smilasaponin, smilax saponins A-C, smiglaside A-E, smitilbin, stigmasterol and taxifolin, and titogenin [159, 160].

### 3.29. *Solanum nigrum* Linn

*Solanum nigrum *Linn. (family: Solanaceae) locally known as “Anaab ud dib” is an annual hairy herb with ovate to oblong leaves abundant in all parts of Saudi Arabia [6]. The plant is a house hold remedy for liver disorders, jaundice and cirrhosis, inflammatory condition, rheumatism, and swollen joints [161–163].

The extracts of whole plant of* S. nigrum *significantly attenuated CCl_4_ [164, 165, 249–251] and thioacetamide [252] induced biochemical (serum AST, ALT, APT, and TB) and histopathological changes in liver. The hepatoprotective action of* S. nigrum* may be attributed to its antioxidant [253] and anti-inflammatory [254] constituents. LD_50_ value of ethanol extract of the fruits of* S. nigrum *in rats was found to be 2 g/kg b.w. [255]. Phytochemical studies on* S. nigrum *showed the presence of glycoalkaloids, glycoproteins, polysaccharides, gallic acid, catechin, protocatechuic acid, caffeic acid, epicatechin, rutin, and naringenin [164, 165].

### 3.30. *Suaeda maritima* Linn

*Suaeda maritima *Linn. (family: Amaranthaceae) locally known as “Sawad” is shrubs with continuous unjoined stems found in western region of Saudi Arabia [6, 256]. The juice of this herb is used for treatment of liver diseases by Arab practitioners [257]. The leaves are also used as remedy for liver, heart, and lipid disorders [166].

The ethanolic extracts of* S. maritima *leaves significantly attenuated concanavalin (a hepatotoxin) induced biochemical (serum AST, ALT, APT, and bilirubin) and histopathological changes in liver [167]. The extract of plant also showed significant antioxidant, anti-inflammatory, antiviral, and antibacterial activities [167, 168] which may contribute to its hepatoprotective activity. It is nontoxic edible plant which is used in salad and as fodder for animals [258]. The LD_50_ of ethanolic extract of* S. maritima* in rats was found to be 3 g/kg b.w. [167]. Phytochemical studies on plant of* S. maritima* showed the presence of alkaloid, flavonoid, sterols, phenolic compounds, and tannins [166–168].

### 3.31. *Tamarix nilotica* Ehrenb Bunge

*Tamarix nilotica *Ehrenb Bunge (family: Tamaricaceae) locally known as “Tarafa” is a green shrub with free distinct blade type leaves found in eastern Najd and northern region of Saudi Arabia [6]. Avicenna has mentioned this plant in his famous book “Canon of medicine” for the treatment of liver, stomach, and inflammatory problems [169–171].

The hydroalcoholic extract of* T. nilotica* flower showed marked hepatoprotective activity against CCl_4_ induced liver injury [171]. Experimental studies also showed highly significant antioxidant [171] and anti-inflammatory [259] activities of* T. nilotica* which may contribute its hepatoprotective activity. No experimental and clinical toxicity of* T. nilotica* has been reported. However plant possesses significant cytotoxicity against some human cancer cell lines [170]. Phytochemical studies on* T. nilotica *showed the presence of flavonoids, tannins, syringaresinol, isoferulic acid, niloticol, 3-hydroxy-4-methoxycinnamaldehyde, methyl and ethyl esters of gallic acid, para-methoxygallic acid, kaempferol, quercetin 3-oglucuronides, 3-o-sulphated kaempferol, 7,4′-dimethyl ether, and free flavonols [170, 171].

### 3.32. *Tephrosia purpurea* Linn

*Tephrosia purpurea *Linn. (family: Fabaceae) locally known as “Ami” is a perennial plant with imparipinnate leaves and grows in southern Hejaz region of Saudi Arabia [6].* T. purpurea* has been used for centuries in traditional system of medicine for the treatment of jaundice, liver, biliary and splenic disease, and inflammatory disorders [172–174].

The hydroalcoholic extract of aerial parts of* T. purpurea *attenuated thioacetamide [175] induced hepatotoxicity in a dose dependent manner suggesting its significant hepatoprotective activity. The extract also showed antioxidant [175] and anti-inflammatory [260] activities, which may contribute to its hepatoprotective activity.* T. purpurea* is well tolerated in rats and produces no toxicity up to the dose of 2000 mg/kg b.w. Chronic administration of* T. purpurea* at doses of 200 and 400 mg/kg b.w. was also found safe in rats [261]. Phytochemical studies on* T. purpurea *have shown the presence of *β*-sitosterol, quercetin, lupeol, rutin, delphinidin chloride, cyanidin chloride, isolonchocarpin, lanceolatins A and B, pongamol, karangin, kangone, 5,7-dimethoxy-8-flavanone, and 2-methoxy-3,9-dihydroxycoumestone [175, 176].

### 3.33. *Teucrium polium* Linn

*Teucrium polium *Linn. (family: Lamiaceae) locally known as “Jaad” is a perennial branched shrub found in northern region, Nefud region, southern Hejaz, East Najd, and eastern province of Saudi Arabia [6].* T. polium* is widely used by the folk-medicine practitioners of Saudi Arabia for the treatment of liver diseases, inflammatory disorders, stomach and intestinal troubles, and rheumatism [177].

The hydroalcoholic extract of aerial part of* T. polium *dose dependently attenuated CCl_4_ [178] and acetaminophen [262] induced biochemical (serum ALT, AST, APT, and total bilirubin) and histological changes in liver. Experimental studies of* T. polium* on cultured hepatocytes also confirmed its strong antioxidant [178, 263] and anti-inflammatory activities [177, 264, 265] which may contribute to its hepatoprotective activity. There is no report on acute toxicity of plants. However chronic administration of high dose of* T. polium* rats showed mild toxicity [266]. One case of severe hepatotoxicity has been reported in a patient following prolonged use of* T. polium *[267, 268]. Phytochemical studies on* T. polium* showed the presence of flavonoids, terpenes including syrapoline, thujene, caryophyllene, cedrol, epi-cadinol, and bisabolene [178–181].

### 3.34. *Trianthema portulacastrum* Linn

*T. portulacastrum* Linn. (family: Aizoaceae) locally known as “Laani” is a fleshy herb with opposite petiolated unequal leaves found in eastern province and southern Hejaz part of Saudi Arabia [6].* T. portulacastrum* is widely used in Arab countries [269], Africa, India, and southeast Asia, for the treatment of jaundice, liver disorders, stomach problem, arthritis, and inflammation [182, 270, 271]. Laboratory investigations on extracts of the plant have demonstrated significant hepatoprotective, antioxidant, diuretic, analgesic, and anticarcinogenic activity [182].

The ethanolic extract of leaves of* T. portulacastrum* significantly attenuated the paracetamol [272], thioacetamide [272], and aflatoxin B [183] induced hepatotoxicity in experimental studies. The extract of* T. portulacastrum* also showed significant antioxidant [273, 274] and anti-inflammatory [275] activities, which may contribute to its hepatoprotective effect. Acute toxicity studies in albino mice suggested that the extract of* T. portulacastrum* was safe even at the dose of 3 g/kg b.w. [276]. Phytochemical studies on* T. portulacastrum *showed the presence of steroids, saponins, flavonoid, coumarins, terpenes, glycosides, tannins, alkaloids, and volatile oil [182, 183].

### 3.35. *Tribulus terrestris* Linn

*Tribulus terrestris *Linn. (family: Zygophyllaceae) locally known as “Darisa” is an annual procumbent herb with compound paripinnate leaves found in eastern Najd and southern Hejaz region of Saudi Arabia [6]. Local bedouin use the plant to treat urinary disorders, impotency, and liver diseases. The seeds of this plant are recommended in hemorrhages, kidney stone, and gout. The fruit is regarded as tonic, diuretic, and aphrodisiac [184].

The aqueous and hydroalcoholic extracts of fruit of* T. terrestris* dose dependently attenuated paracetamol [277] and ferrous sulphate [278] induced liver damage. Two compounds (tribulusamides A and B), isolated from the fruits of* T. terrestris* significantly, protected cultured hepatocytes against* D*-galactosamine induced toxicity [279].* T. terrestris* has also been reported to possess antioxidant [280] and anti-inflammatory [281] activities which may contribute to its hepatoprotection. According to some reports grazing on* T. terrestris *caused hepatorenal syndrome and neurotoxicity in goats and sheep [282, 283]. Nephrotoxicity has also been reported in patient following chronic use of* T. terrestris *[284]. The extract also showed antispasmodic activity in rats [285]. Phytochemical studies on* T. terrestris* showed the presence of Tribulusamides A and B, tigogenin, neotigogenin, terrestrosid F, and gitonin [185].

## 4. Conclusion

Ancient classical literature and ethnomedical survey among local population clearly suggest that herbal drugs have been extensively used in Arab traditional medicine for the treatment of liver diseases. In this review we present the scientific appraisal of 35 herbal drugs used in Saudi traditional medicine for the treatment of liver disorders. The effect of herbs against hepatotoxin induced liver injury (based on biochemical markers and histopathological findings) has been summarized. Besides reviewing hepatoprotective efficacy and possible mechanism of action of these plant drugs, the available data on phytochemical constituents and their toxic untoward effects have been presented. Although the meta-analysis of available scientific literature on hepatoprotective activity of the herbs to a great extent substantiates folkloric claims about the usefulness of these botanicals to treat chronic liver diseases, the data regarding randomized clinical trials, safety studies, and quality control of these herbs is far from satisfactory.

**Figure 1:**
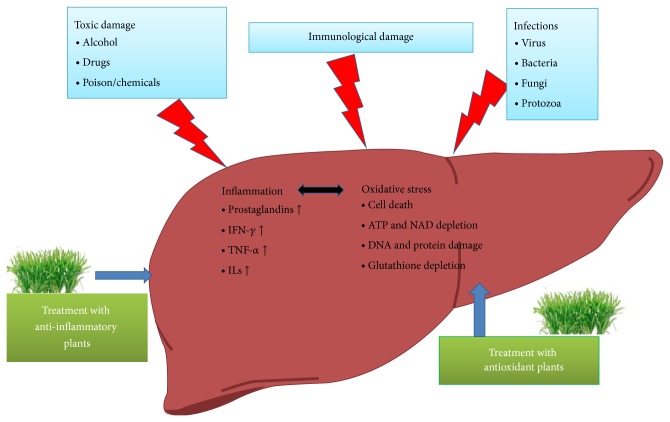
Anti-inflammatory and anti-oxidant herbs protect liver against variety of toxins and injurious stimuli by restoring the oxidative stress related liver damage and inflammatory cytokines.

One of the noteworthy findings in this review is that the majority of hepatoprotective plants showed antioxidant and anti-inflammatory activities. The mechanism of hepatic injury invariably involves peroxidation of hepatocyte membrane fatty acids causing destruction of the cells and their intracellular organelles. According to the recent reports oxidative stress plays a pivotal role in the initiation and progression of hepatic damage following insult to a variety of hepatotoxins. The role of oxidative stress in viral hepatitis and autoimmune related liver diseases has been extensively documented. Moreover hepatotoxic chemicals damage liver cells primarily by producing reactive species which form covalent bond with the lipid moiety of the hepatic cell membranes (Figure 1).

Due to extensive exposure to hazardous chemicals, sometimes the free radicals generated are so high that they overpower the natural defensive system leading to hepatic damage. The drugs/chemicals with antioxidant properties such as Vitamin E and silymarin have been shown to protect against toxin induced hepatotoxicity. On the other hand inflammation is a key event in hepatotoxin induced liver damage. The toxins directly or through oxidative stress mechanism may trigger inflammatory response in the liver, which is evident from a significant increase in the proinflammatory cytokines including TNF*α* and IL6 and hepatocyte inflammation. Majority of hepatoprotective herbs have been shown to suppress oxidative stress and inflammation.

**Figure 2:**
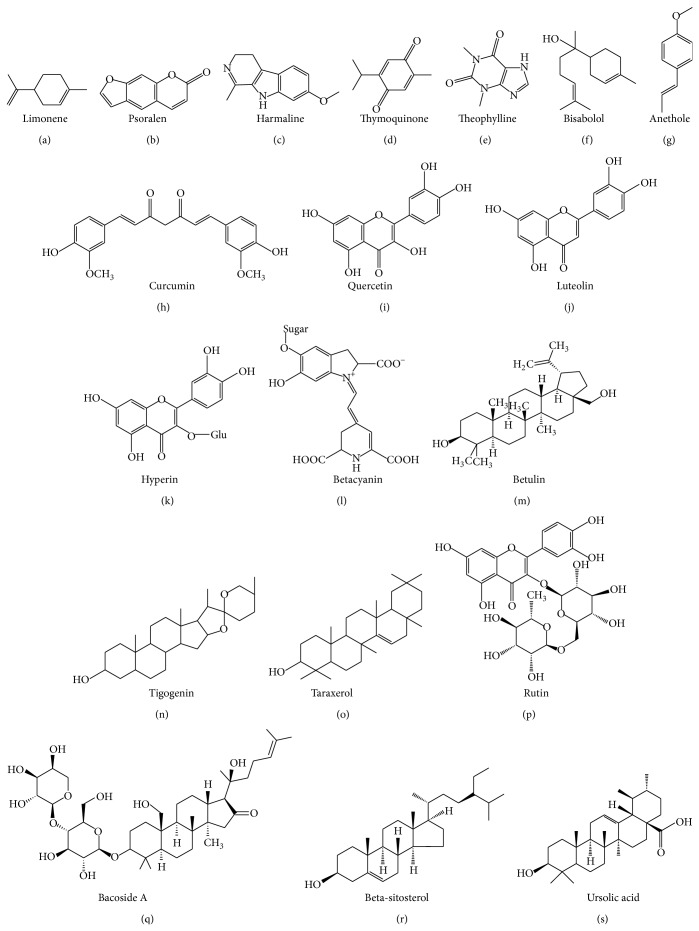
Chemical structure of some of the hepatoprotective phytoconstituents.

Our survey and published reports clearly suggest that medical plants used in traditional medicine are rich sources of medicinally active chemical constituents such as phenols, coumarins, lignans, terpenoids, carotenoids, glycosides, flavonoids, organic acids, alkaloids, and xanthene. Some of the purified phytomolecules isolated from these plants have also been shown to possess potent hepatoprotective activity (Figure 2).

Further investigation into the lead molecules that may produce better, safe, and effective therapeutic effects is warranted to overcome the pharmaceutical imbalance between remedies that protect the liver and drugs that induce hepatotoxicity. Moreover quality control of herbal drugs and randomized controlled clinical trials will further validate the evidenced based herbal therapy for the treatment of liver diseases.

## Figures and Tables

**Table 1:** Saudi herbal drug with hepatoprotective activity.

Plant name	Traditional uses	Chemical constituent	Reference
*Apium graveolens *	Liver and spleen disorders, jaundice, rheumatism, gout, and inflammatory diseases	Limonene, p-dimethyl styrene, n-pertyl benzene, caryophyllene, a-selinene, n-butyl phthalide, and sedanenolide	[7–9, 13]
*Artemisia scoparia *	Jaundice and liver disorders	Hyperin, eupafolin, pedalitin, 5,7,2′,4′-tetrahydroxy-6,5′-dimethoxyflavone, camphor, and 1,8-, and beta-caryophyllene	[16, 17, 26]
*Bacopa monnieri *	Jaundice, liver diseases, spleen disorders, and digestive problems	Brahmine, bacosides-a, nicotine, herpestine, d-mannitol, and hersaponin	[27, 28, 34]
*Balanites aegyptiaca *	Jaundice, liver disorders, and spleen problems	Quercetin 3-glucoside, quercetin-3-rutinoside; 3-glucoside, 3-rutinoside, 3-7-diglucoside, and 3-rhamnogalactoside	[35, 41]
*Beta vulgaris *	Spleen, liver problems, and inflammatory disorders	Betacyanins, betaxanthins, oxalic acid, and ascorbic acid	[42–44, 47]
*Boerhavia diffusa *	Jaundice and other liver diseases, internal inflammation, gall bladder problem, and spleen disorders	Punarnavine, boeravinones, flavonoids, amino acids, lignans, and tetracosanoic, esacosanoic, stearic, and ursolic acids	[48]
*Camellia sinensis *	Obesity/weight loss, arthritis, and other inflammatory conditions	Caffeine, theophylline, and theobromine	[54, 62]
*Clitoria ternatea *	Liver diseases	Taraxerol, taraxerone, ternatins, flavonoids, saponins, and tannins	[63, 64, 66, 69]
*Commiphora opobalsamum *	Stomach, jaundice, liver diseases, joint pain, and inflammatory disorders	Flavonoids, saponins, volatile oil, sterol, and/or triterpenes	[72, 73]
*Curcuma longa *	Loss of appetite, jaundice, liver problems, gall bladder disorders, and arthritis	Curcumin, demethoxycurcumin, and bis-demethoxycurcumin	[76–78]
*Eruca sativa *	General tonic, liver, and intestinal disorders	Glucosinolates, quercetin, and erucic acid	[82–84, 86]
*Ficus carica *	Liver disease, stomach ailments, digestive problems, obesity, and inflammatory diseases	Psoralen, mucilages, flavonoids, vitamins, nicotinic acid, tyrosine, ficusin, bergaptene, stigmasterol, taraxasterol, beta-sitosterol, rutin, and sapogenin	[89–93, 97]
*Grewia mollis *	Liver disease, abdominal problems, arthritis, and inflammatory conditions	Luteolin, tetrahydroxyflavone, 7*β*-hydroxy-23-enedeoxojessic acid, 7*β*-hydroxy-23-deoxojessic acid, *β*-sitosterol, and *β*-sitosterol-3-o-glucoside	[99–102]
*Grewia tenax *	Liver disorders, jaundice, and inflammatory condition	Betulin, triacontan-1-ol, *α*-amyrin, *β*-amyrin, *β*-sitosterol, lupenne, erythrodiol, and tetratriacont-21-ol-12-one	[4, 104, 105]
*Haloxylon salicornicum *	Jaundice, gall bladder stones, liver diseases, digestive disorders, inflammatory disorder, and joint diseases	Ursolic acid, 7-hydroxy-4-triacontanone, 24-hydroxy-4-octacosanone, 1-triacontanol, *β*-amyrin, 24-ethylcholesta-3,5-diene, 24-nor-12-ursene, *β*-sitosterol, and *β*-sitosterol	[106–111]
*Hypericum perforatum *	Jaundice, liver diseases, gall bladder stones, rheumatoid arthritis, and inflammatory conditions	Rutin, hypericin, pseudohypericin, hyperforin, adhyperforin, quercetin, hyperoside, campferol, myricetin, amentoflavone, i3, kielcorin, and norathyriol	[112–115]
*Juniperus procera *	Liver disease, jaundice, digestive problems, inflammatory diseases, and ulcers	*β*-peltatin a, methyl ether and deoxypodophyllotoxin, and totarol	[115–117]
*Lepidium sativum *	Jaundice, liver problems, spleen diseases, gastrointestinal disorders, arthritis, and inflammatory disorders	Alkaloids, saponins, anthracene glycosides, carbohydrates, proteins, amino acids, flavonoids, and sterols	[53, 118]
*Moringa oleifera * (seed oil)	Liver disease, lipid disorders, arthritis, and inflammatory disorders	*β*-carotene, protein, and vitamin c	[119–122]
*Nigella sativa *	Liver tonics, digestive, anti-inflammatory, immunostimulant, and remedy for jaundice	Thymoquinone, thymohydro quinine, dithymoquinone, p-cymene, carvacrol, and 4-terpineol	[123–125]
*Peganum harmala *	Jaundice, digestive disorders, liver disease, and arthritis	Harmaline, harmine, harmalol, and tetrahydroharmine	[126–131]
*Pergularia daemia *	Jaundice, liver diseases and inflammatory disorders	Cardenolides, alkaloid, saponins, triterpenes, and steroidal compounds	[132–135]
*Petroselinum crispum *	Liver diseases, constipation, flatulence, jaundice, colic pain and rheumatism	Flavone glycosides	[136–140]
*Phyllanthus maderaspatensis *	Emetic and purgative, constipation, digestion and abdominal pain liver disorders, rheumatism and inflammatory diseases	Essential oil, mandarin, mucilage, and *β*-sitosterol	[141–144]
*Pimpinella anisum *	Digestive, carminative, antispasmodic and for liver disorders	Trans-anethole and palmitic and oleic acids	[145–147]
*Portulaca oleracea *	Liver disorders, gastrointestinal problems and inflammatory disorders	Omega-3 fatty acids, alpha-linolenic acid, and vitamins a, b, and c	[82, 148–151]
*Rhazya stricta *	Stomach problems, liver diseases and inflammatory disorders	Akuammidine, bhimberine, rhazimol	[36, 152–155]
*Smilax regelii *	Liver diseases, arthritis and inflammatory conditions	Saponins flavonoids, tannins, sterols, and triterpenes	[156–160]
*Solanum nigrum *	Liver disorders, jaundice and cirrhosis, inflammatory disorders, rheumatism and swellen joints	Glycoalkaloids, glycoproteins, polysaccharides, gallic acid, catechin, protocatechuic acid, caffeic acid, epicatechin, rutin, and naringenin.	[161–165]
*Suaeda maritima *	Liver, heart and lipid disorders	Alkaloid, flavonoid, and tannins	[166–168]
*Tamarix nilotica *	Liver, stomach and inflammatory problems	Kaempferol, syringaresinol, isoferulic acid, niloticol, 3-hydroxy-4-methoxycinnamaldehyde, methyl and ethyl esters of gallic acid, para-methoxygallic acid, quercetin 3-oglucuronides, 3-o-sulphated kaempferol, and 7,4′-dimethyl ether	[169–171]
*Tephrosia purpurea *	Jaundice, liver, biliary and splenic disease, and inflammatory disorders	*β*-sitosterol, quercetin, lupeol, rutin, delphinidin chloride, cyanidin chloride, isolonchocarpin, lanceolatins a and b, pongamol, karangin, kangone, 5,7-dimethoxy-8-flavanone, 2-methoxy-3,9-dihydroxycoumestone	[172–176]
*Teucrium polium *	Liver diseases, inflammatory disorders, stomach and intestinal troubles and rheumatism	Caryophyllene, cedrol, a-epi-cadinol, and e-g-bisabolol	[177–181]
*Trianthema Portulacastrum *	Liver diseases and pain	Flavonoid, steroids, fats, terpenes, carbohydrates, tannins, and alkaloids	[182, 183]
*Tribulus terrestris *	Tonic, diuretic, and aphrodisiac	Tigogenin, neotigogenin, terrestrosid F, and gitonin	[184, 185]
